# The Influence of Viable Cells and Cell-Free Extracts of *Lactobacillus casei* on Volatile Compounds and Polyphenolic Profile of Elderberry Juice

**DOI:** 10.3389/fmicb.2018.02784

**Published:** 2018-11-20

**Authors:** Annalisa Ricci, Alessia Levante, Martina Cirlini, Luca Calani, Valentina Bernini, Daniele Del Rio, Gianni Galaverna, Erasmo Neviani, Camilla Lazzi

**Affiliations:** ^1^Department of Food and Drugs, University of Parma, Parma, Italy; ^2^Department of Veterinary Science, University of Parma, Parma, Italy

**Keywords:** elderberry juice, *Lactobacillus casei*, volatile compounds, polyphenols, microbial growth, cell-free extracts

## Abstract

In this study, four strains of *Lactobacillus casei*, as viable cells or cell-free extracts (CFE), were added to elderberry juice in order to evaluate their effect on phenolic and aromatic profile. Two of them were able to grow in juice while the others showed zero-growth. The same strains were lysed and added as extracts in elderberry juice. Multivariate statistical analysis show a separation among samples containing growing cells, non-growing cells, CFE, highlighting the particularities of specific strains. Juices added with CFE presented the highest amount of esters. The strains showing growth phenotype cause an increase of phenyllactic acids. The highest concentration of volatile compounds, particularly of alcohols, terpenes and norisoprenoids (responsible for typical elderberry notes) was observed in samples with strains showing zero-growth. Moreover, a significant increase in anthocyanin content was observed in these samples, suggesting the possible use of *Lactobacillus* for increasing specific molecules, even for non-multiplying bacterial cell. Considering that this is the first study concerning the use of non-growing cells in fruit juice, the potential of strains is still to be explored and it may have a significant technological application in the development of a microbial collection useful for fruit juice industry.

## Introduction

Lactic acid bacteria (LAB) fermentation is an ancient technology of bio-preservation that in the last decade has been extensively applied to vegetable and fruit to increase their consumption. Moreover, following the fermentation, the nutritional value and different organoleptic traits, which are generally compromised during other manufacturing processes, can be improved (Ricci et al., 2018). As for other fermented food products, the selection of strains able to perform the conversion of substrate is essential to tailor specific functional or sensorial properties. Generally, selection of LAB strains for these applications implies: (i) the ability of growth, (ii) the synthesis of aroma compounds or precursor that can improve the sensorial features, (iii) the production of metabolic end-products with nutritional value (Di Cagno et al., 2013). Cell adaptation and replication may depend upon polyphenols, which are abundant in fruits, and exert an inhibitory activity by damaging cytoplasmic membranes and cell walls, but could be converted by microbial enzymes into different metabolites, less toxic to cells and perhaps endorsed with other bioactivities (Huynh et al., 2014; Filannino et al., 2018). New metabolites could be produced through different bioconversion pathways, such as glycosylation, deglycosylation, ring cleavage, methylation, glucuronidation, and sulfate conjugation (Huynh et al., 2014). To date, the metabolism of phenolics has been investigated, among LAB, mainly for *Lactobacillus plantarum* (Rodríguez et al., 2009; Filannino et al., 2018). Even if *L. plantarum* is the LAB species most used to ferment fruits (Rodríguez et al., 2009), a recent study reported that also other species, as *Lactobacillus casei* and *Lactobacillus rhamnosus*, may be used in fermentation processes (Ricci et al., 2018). These closely related species belonging to the *Lactobacillus casei* group and representing the most widely researched and applied probiotic species of lactobacilli (Hill et al., 2018). In particular, fermentation of elderberry juice with dairy strains was found to be useful to enhance the typical aroma of elderberry (mainly linked to terpenes and alcohols), thus opening new opportunities in the selection of LAB for fruit fermentation (Ricci et al., 2018).

In other fermented foods the biochemical change of substrate relies not only on the action of metabolically active microbial cells but also on the activity of cytoplasmatic enzymes released after cell lysis (Smid and Kleerebezem, 2014).

Indeed, the action of intracellular enzymes has a great relevance in aroma formation (Lazzi et al., 2016). The effect of microbial cell lysis (in LAB, yeasts and molds) or the addition of cell-free extracts was described by different authors in various fermented foods, such as dairy products, sausages and wine. Thanks to specific release of enzymes from the broken cells, the acceleration of ripening and/or the generation of volatile compounds occur (Lortal and Chapot-Chartier, 2005; Bolumar et al., 2006; Styger et al., 2011), but to our knowledge this topic has never been explored in fruits matrices.

Based on these premises, this study focused on the effect on viable cells and CFE added to elderberry juice. To this aim four strains of *L. casei*, isolated from dairy products, considered both as viable cells and CFE, were individually added to commercial elderberry juice in order to evaluate the differences among samples based on phenolic fraction and aromatic profile.

## Materials and methods

### Chemicals

Cyanidin-3-*O*-rutinoside (aka keracyanin), cyanidin-3-*O*-glucoside (aka kuromanin) and luteolin were purchased from Extrasynthese (Genay Cedex, France). 3-*O*-caffeoylquinic acid (aka neochlorogenic acid), 4-*O*-caffeoylquinic acid (aka cryptochlorogenic acid), 5-*O*-caffeoylquinic acid (aka chlorogenic acid), kaempferol, quercetin-3-*O*-rutinoside hydrate (aka rutin hydrate), quercetin dihydrate, DL-3-phenyllactic acid, 3,4-dihydroxybenzoic acid (aka protocatechuic acid), caffeic acid and toluene were purchased from Sigma-Aldrich (St. Louis, MO, USA). DL-*p*-hydroxyphenyllactic acid was from Santa Cruz Biotechnology (Dallas, TX, USA). HPLC-grade acetonitrile was from Sigma-Aldrich, while HPLC-grade water and LC-MS grade formic acid were purchased from VWR International (Milan, Italy).

### Bacterial strains

Four strains of *L. casei* belonging to the Food and Drug Department, University of Parma (Italy) were used as adjunct in elderberry juice. *L. casei* 2057, isolated from Grana Padano cheese and *L. casei* 2306, 2240, and 2246 from Parmigiano Reggiano cheese were maintained as frozen stocks (-80°C) in de Man Rogosa Sharpe (MRS) medium (Oxoid, Milan, Italy) supplemented with 15% glycerol (v/v). The cultures were propagated three times with about 3% (v/v) inoculum in MRS and incubated in anaerobiosis (AnaeroGen, Oxoid) at 37°C for 15 h.

### Preparation of viable cells and CFE

Viable cells to be added to elderberry juices were prepared cultivating the strains until the late exponential phase (ca. 15 h), harvesting the cell by centrifugation (10,000 × g for 10 min at 4°C), washing twice with Ringer's solution (Oxoid, Milan, Italy), and finally re-suspending in sterile distilled water to a final concentration of 9.0 Log CFU/mL. CFE from the same strains were prepared by sonication of microbial cultures using SONOPULS Ultrasonic homogenizers (Bandelin, Germany) In brief, three cycles of sonication at 12 W for 30 s, with 60 s of pause between the cycles, were carried out. MRS plate agar count was performed in duplicate to verify the consistency of sonication. The efficacy of sonication was calculated as follow: E = 100- (n_t_/n_0_)^*^100 where n_0_ = CFU/mL of *L. casei* cultures and n_t_ = CFU/mL of *L. casei* cultures after sonication treatment. The CFE were separated from the membrane residues by subsequent centrifugation at 10,000 × g for 15 min at 4°C (Centrifuge 5810 R, Eppendorf). The supernatant was sterilized by filtration and the absence of growth was evaluated in TSB 0.6% YE (tryptone soya broth, yeast extract) after incubation at 37°C for 48 h. All the samples were conserved at −80°C. Moreover, the protein content of CFE were determined by fluorometric assay, using Qubit protein assay kit and the measurements were carried out with Qubit Fluorometer (Invitrogen, Thermo Fisher Scientific, Massachusetts, USA).

### Elderberry juice added with *l. casei*

A commercial pasteurized non-filtered elderberry juice was added with viable cells and CFE of *L. casei*. Viable cells of *L. casei* 2057, 2306, 2240, 2246, prepared as described previously, were inoculated into elderberry juice in order to reach 7 Log CFU/mL. The juice was fermented at 37°C for 48 h. Microbial counts before and after fermentation were performed in triplicate using MRS Agar medium (Oxoid, Italy) and incubating at 37°C for 48–72 h, under anaerobiosis. Elderberry juice was also added with CFE from the same strains, incubated at 37°C for 48 h and after this period the absence of cultivable cells was evaluated using MRS plate count agar. The eight experiments were carried out in triplicate and elderberry juice not added of *L. casei* was incubated at 37°C for 48 h and used as control. All the samples were maintained at −80°C until the analysis.

### Characterization of volatile profile of elderberry juices with viable cells and CFE of *L. casei*

The volatile profile of elderberry juice samples added with viable inocula and CFE of *L. casei*, incubated for 48 h at 37°C was fully characterized by means of HS-SPME/GC-MS technique following the protocol reported by Ricci et al. (2018). Briefly, 2 mL of elderberry juice were placed in a 20 mL glass vial adding 5 μL of an aqueous toluene standard solution (100 μg/mL in 10 mL). Head space solid phase micro-extraction was performed for 30 min at 40°C after 15 min of equilibration time. For each analysis a SPME fiber coated with 50/30 μm of Divinylbenzene–Carboxen–Polydimethylsiloxane (DVB/Carboxen/PDMS) was used (Supelco, Bellefonte, PA, USA). Before the analyses, the fiber was conditioned by insertion into the GC–MS injector at 230°C for 2 min, analogously, the desorption of volatiles was accomplished exposing the fiber into the GC injector for 2 min at 230°C. GC–MS analyses were performed on a Thermo Scientific Trace 1300 gas chromatograph coupled to a Thermo Scientific ISQ single quadrupole mass spectrometer equipped with an electronic impact (EI) source. All samples were injected in splitless mode, maintaining the valve closed for 2 min. Helium was used as carrier gas with a total flow of 1 mL/min. The separation was performed on a SUPELCOWAX 10 capillary column (Supelco, Bellefonte, PA, USA; 30 m × 0.25 mm × 0.25 μm) at programmed temperature starting from 50°C for 3 min, increasing of 5°C per min to reach 200°C and maintaining this final temperature for 12 min. The transfer line temperature was 250°C. The signal acquisition mode was full scan (from 41 m/z to 500 m/z). The main volatile compounds of elderberry juices were identified on the basis of their mass spectra compared with the library (NIST 14) mass spectra. To obtain a more confident identification, the linear retention indices (LRIs) were calculated on the basis of the retention times of a solution of C8–C20 alkanes analyzed under the same conditions applied for sample analyses. The semi-quantification of all detected gas-chromatographic signals was performed on the basis of the use of an internal standard (toluene).

### Phenolic compounds analysis

All the juices added with *L. casei* and the control were analyzed with an Accela UHPLC 1250 equipped with a linear ion trap-mass spectrometer (MS) (LTQ XL, Thermo Fisher Scientific Inc., San Jose, CA, USA) fitted with a heated-electrospray ionization probe (H-ESI-II; Thermo Fisher Scientific Inc., San Jose, CA, USA). Analyte separation was performed using an Acquity UPLC HSS T3 (2.1 × 100 mm) column coupled with a pre-column Acquity UPLC HSS T3 VanGuard (2.1 × 5 mm) (Waters, Milford, MA, USA). The injection volume was 5 μL, the autosampler temperature was set at 10°C and column compartment temperature was set at 40°C. For the determination of phenolic profile was followed the protocol reported by Zaupa et al. (2015) with some modification. Briefly, acetonitrile (phase A) and water (phase B) both acidified with formic acid (0.1% v/v) were used as eluents applying a gradient of elution. In particular, the initial conditions were set at 95% B and 5% A and maintained for 0.5 min, then the phase A turned up at 51% in 9 min. After this step, at 9.5 min phase A was increased at 80%, the column was flashed under these conditions for 2 min and then, at 12 min, initial proportions were re-established. The total run time was 17 min at a flow rate of 0.3 mL/min.

The anthocyanin detection was carried out in positive ionization mode, with a capillary temperature of 275°C, and a source heater temperature of 300°C. The sheath gas (nitrogen) flow was 40 units, while auxiliary gas (nitrogen) was equal to 5 units. The source voltage was 4.5 kV, while the capillary and tube lens voltages were 20 and 95 V, respectively. Untargeted preliminary analyses were carried out in order to evaluate differences in the anthocyanin profile among the investigated samples. In detail, the mass spectrometer worked in full scan mode, data-dependent MS^3^ scanning from m/z 220–900, with CID equal to 30. Once performed the identification, the anthocyanins have been quantified in Selective Reaction Monitoring (SRM) mode using a Collision Induced Dissociation (CID) equal to 30. Cyanidin-3-*O*-glucoside was quantified through its authentic standard compound, while cyanidin-*O*-sambubioside, cyanidin-*O*-sambubioside-*O*-hexoside and cyanidin-3-*O*-rutinoside were quantified by using authentic standard of cyanidin-3-*O*-rutinoside.

The other polyphenolic compounds were analyzed in negative mode, applying the same capillary and source temperatures used before. The sheath gas (nitrogen) flow was 40 units, while auxiliary gas (nitrogen) was equal to 5 units. The source voltage was 4 kV, while the capillary and tube lens voltages were −33 and −98 V, respectively. Untargeted preliminary analyses were carried out in order to evaluate differences in the polyphenolic profile, as cited above for anthocyanin profile. In detail, the mass spectrometer worked in full scan mode, data-dependent MS^3^ scanning from m/z 100–2,000, with CID equal to 35. Once performed the identification through untargeted analyses, the aglycone flavonoids have been quantified in SIM mode, while the other negatively charged polyphenols were quantified in SRM mode using a CID equal to 35. Pure helium gas was used for CID. Data processing was performed using Xcalibur 2.2 software from Thermo Fisher Scientific Inc. (San Jose, CA, USA). Where possible, polyphenols were quantified trough external calibration curves by using the reference standard compound. When such standards were not available, polyphenols were quantified using a structurally related compound. In detail, isorhamnetin has been quantified as quercetin dihydrate equivalents, coumaroylquinic acid I and coumaroylquinic acid II were quantified as 3-*O*-Caffeoylquinic acid and 5-*O*-Caffeoylquinic acid, respectively. Quercetin-3-*O*-glucoside, isorhamnetin-*O*-hexoside, kaempferol-*O*-rutinoside, isorhamnetin-*O*-rutinoside and quercetin-*O*-rutinoside-*O*-hexoside were quantified as rutin hydrate equivalents.

### Chemometrics analysis

One way ANOVA was used to compare the different results obtained in juices added with viable and CFE *L. casei* strains, performed with IBM SPSS statistics package v. 21 (IBM Corp., Armonk, NY, USA). To evaluate the normal distribution for every group of independent samples Shapiro-Wilk test was used; multiple comparisons were performed with Tukey's test, differences were considered statistically significant when *p* < 0.05. Data obtained from the analyses, such as volatile compounds amounts and polyphenols concentration were analyzed by Principal Component Analysis (PCA) using the packages *ade4* and *FactoMineR* on R environment (http://www.r-project.org). The principal coordinates were selected depending on their weight on each of the principal components, and a threshold value was set at ± 0.75 (Table S1). Volatile compounds and polyphenols concentrations were Z-transformed and used to build heatmaps, by using the *cim* function in *mixOmics* package; radarplot graphs were built using package *fmsb*, both running on R software v. 3.4.1.

## Results

### Growth of *L. casei* in elderberry juice

The elderberry juice was added, singularly, of four cultures containing viable cells of *L. casei* (2057, 2306, 2240, and 2246) and of the respective CFE. The growth ability of the four viable strains was evaluated after 48 h of incubation by plate count in MRS agar, revealing two different behaviors. Strains 2057 and 2306 shown zero-growth, meaning that cell densities remained unchanged from the original inocula (about 7 Log CFU/mL). On the other hand, strains 2240 and 2246 grew of about 2 Log CFU/mL. During the incubation of the started juices the pH only moderately decreased from an initial value of 3.84 ± 0.07 to 3.73 ± 0.05 (mean value). Unstarted juice (control sample) didn't show microbial growth after 48 h.

### *L. casei* lysis

The efficacy of sonication in *L. casei* cellsresulted equal to 99%. To obtain a sterile sample the absence of bacterial growth was evaluated in TSB added with 0.6% of yeast extract after filtration step. In order to analyse the protein content of CFE, fluorometric assay was used. The protein content obtained was 0.81 ± 0.10 mg/mL (zero-growth strains) and 2.59 ± 0.05 mg/mL (grow strains) according with cellular concentration. CFE was added in elderberry juice to obtain comparable concentrations with viable cells (9 Log CFU/mL, grow strains, 2240 and 2246; 7 Log CFU/mL, zero-growth strains, 2057 and 2306) and the absence of bacterial growth was tested after 48 h of incubation.

### Global view: PCA and heatmap of volatile compounds and polyphenols

**Figure 1:**
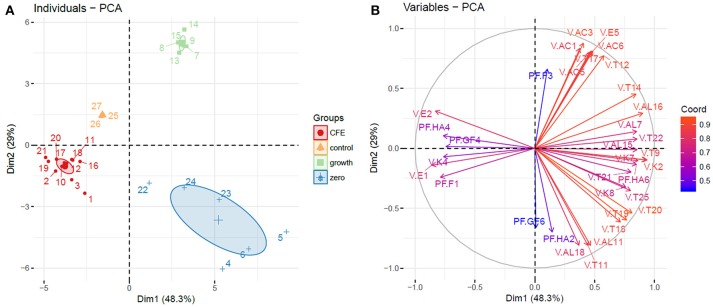
Principal component analysis (PCA). **(A)** Individuals plot, samples are represented as follows: 1–3, *L. casei* 2057 CFE; 4–6, *L. casei* 2057 zero growth; 7–9, *L. casei* 2240 growth; 10–12, *L. casei* 2240 CFE; 13–15, *L. casei* 2246 growth; 16–18, *L. casei* 2246 CFE; 19–21, *L. casei* 2306 CFE; 22–24, *L. casei* 2306 zero growth. Ellipses are drawn at a confidence level of 0.95. **(B)** Variables plot based on volatile compounds and polyphenols with higher influence on elderberry juices characterization, variables are colored according to their weight on the PCA dimensions. Abbreviations are the same as in Table 2 for volatile compounds (V.) and Table 4 for polyphenols (PF.).

Alcohols, terpenes and norisoprenoids, acids, ketones and esters (67 compounds) were the main volatiles detected and semi-quantified in elderberry juices added with viable cells, CFE and control (Tables 1, 2). Phenolic acids and flavonoids (Tables 3, 4) were also identified and quantified resulting in a total of 23 compounds. PCA considering the identified volatiles and polyphenols was performed to highlight the differences among the samples analyzed. Considering the 90 variables, the total variance explained by PCA accounted to 64.8% and a good clustering of the samples was obtained according to the growth profile of the viable cells, either growing or zero-growing, and of juices added with CFE (data not shown). Nevertheless, due to the high number of variables used for the classification, 34 of them belonging to volatile compounds (acids, alcohol, esters, ketones, terpenes) and polyphenols (anthocyanins, flavonol glycosides, hydroxycinnamic acids) were selected according to their contribution either to dimension 1 or dimension 2 of the PCA (Table S1). The new PCA analysis explains 77.3% of the variance, with PC1 and PC2 describing 48.3 and 29% of variance, respectively (Figures 1A,B).

**Table 1:** Assignment of GC-MS signals and their relatives flavor notes.

**Peak no**.	**Identification**	**Flavor note**	**LRI**	**Identification method**	**References**
1	Acetone	Ethereal, apple, pear	776	MS	–
2	Ethyl acetate	Ethereal, fruity	855	MS + LRI	Dall'Asta et al., 2011
3	Ethanol	Strong, alcoholic	903	MS + LRI	Goodner, 2008
4	Diacetyl	Strong, butter	973	MS + LRI	Ferreira et al., 2001
5	Methyl isovalerate	Strong, apple, pineapple	1,020	MS + LRI	Sanz et al., 2001
6	Terpene not specified	–	1,059	MS	–
7	Ethyl isovalerate	Fruity, sweet, apple	1,065	MS + LRI	Ferreira et al., 2001
8	Isopentyl acetate	Sweet, fruity, banana	1,117	MS + LRI	Ferreira et al., 2001
9	3-Carene	Sweet, citrus, terpenic	1,128	MS + LRI	Chung et al., 1993
10	Myrcene	Peppery, spicy	1,143	MS + LRI	Kaack et al., 2005
11	Limonene	Citrus	1,175	MS + LRI	Cirlini et al., 2012
12	Eucalyptol	Eucalyptus	1,197	MS + LRI	Chisholm et al., 2003
13	Isoamyl alcohol	Alcoholic, whiskey	1,220	MS + LRI	Dall'Asta et al., 2011
14	γ-terpinene	Oily, woody, terpenic, tropical	1,227	MS + LRI	Cirlini et al., 2012
15	*m*-cymene	–	1,258	MS + LRI	Cavalli et al., 2003
16	Terpinolene	Fresh, woody, sweet, citrus	1,264	MS + LRI	Choi, 2003
17	Isoamyl isovalerate	Sweet, fruity, green	1,285	MS	–
18	Acetoin	Sweet, buttery, creamy, dairy	1,300	MS + LRI	Bianchi et al., 2007
19	2,3-Octanedione	Dill, cooked, broccoli	1,322	MS + LRI	Bianchi et al., 2007
20	2-Penten-1-ol	Green	1,325	MS + LRI	Pennarum et al., 2002
21	(*E*)-2-Hexenyl-acetate	Green, fruity	1,329	MS + LRI	Jorgensen et al., 2000
22	Sulcatone	Citrus	1,336	MS + LRI	Chung et al., 1993
23	2,3,4,5-Tetramethyl-2-cyclopenten-1-one	–	1,350	MS	–
24	Hexanol	Herbal	1,354	MS + LRI	Cirlini et al., 2012
25	(*E*)-3-Hexen-1-ol	Green, leafy	1,365	MS + LRI	Bianchi et al., 2007
26	(*Z*)-3-Hexen-1-ol	Green, leafy	1,386	MS + LRI	Bianchi et al., 2007
27	Herbal ketone	Fresh, sweet, green, weedy	1,388	MS	–
28	3-Octanol	Earthy, mushroom	1,392	MS	–
29	(*E*)-2-Hexen-1-ol	Fruity, green, leafy	1,407	MS + LRI	Bianchi et al., 2007
30	α-Ionene	–	1,437	MS	–
31	*cis*-Linalool oxide	–	1,442	MS + LRI	Cirlini et al., 2012
32	1-Octen-3-ol	Earthy	1,449	MS + LRI	Cirlini et al., 2012
33	Heptanol	Green	1,455	MS + LRI	Cirlini et al., 2012
34	Acetic acid	Sharp, pungent, vinegar	1,470	MS + LRI	Bianchi et al., 2007
35	β-Linalool	Floral	1,547	MS + LRI	Cirlini et al., 2012
36	Octanol	Waxy, green, orange	1,555	MS + LRI	Dall'Asta et al., 2011
37	β-Caryophyllene	Sweet, woody	1,588	MS + LRI	Bianchi et al., 2007
38	2-Undecanone	Waxy, fruity, creamy	1,598	MS + LRI	Bianchi et al., 2007
39	Hotrienol	Sweet, tropical	1,609	MS + LRI	Kaack et al., 2005
40	6-Methyl-heptanol	–	1,612	MS	–
41	Dihydro-citronellol	Floral	1,617	MS + LRI	Umano et al., 1999
42	6-Methyl-octanol	–	1,626	MS	–
43	Nonanol	Fresh, fatty, floral	1,657	MS + LRI	Dall'Asta et al., 2011
44	2-Furanmethanol	Alcoholic, chemical, caramellic, bready	1,665	MS + LRI	Dall'Asta et al., 2011
45	Isovaleric acid	Stinky feet, cheese	1,675	MS + LRI	Ferreira et al., 2001
46	α-Terpineol	Pine, terpene, lilac	1,695	MS + LRI	Cullere et al., 2004
47	Cadina-1(10)-4-diene	–	1,751	MS	–
48	β-damascenone “like”	Woody, sweet, fruity, earthy	1,758	MS	–
49	β-citronellol (R)	Floral	1,763	MS +LRI	Ferreira et al., 2001
50	Terpene not specified	–	1,773	MS	–
51	Methyl salicylate	Wintergreen mint	1,778	MS + LRI	Kaack et al., 2005
52	Phenethyl acetate	Floral, rose, sweet, honey	1,787	MS + LRI	Ong and Acree, 1999
53	*cis*-Geraniol	Sweet, floral, fruity, rose	1,798	MS + LRI	Nishimura, 1995
54	2-Tridecanone	Fatty, waxy, dairy, milky	1,806	MS	–
55	β-damascenone	Woody, sweet, fruity, earthy	1,820	MS + LRI	Valim et al., 2003
56	*trans*-Geraniol	Sweet, floral, fruity, rose	1,845	MS + LRI	Goodner, 2008
57	Caproic acid	Sour, fatty, sweaty, Cheesy	1,855	MS + LRI	Ferreira et al., 2001
58	2- Phenylmethanol	Floral	1,879	MS + LRI	Chung et al., 1993
59	2-Phenylethanol	Floral	1,914	MS + LRI	Ferreira et al., 2001
60	Caproic acid like	–	1,953	MS	–
61	Dodecanol	earthy, soapy, waxy, Fatty	1,963	MS + LRI	flavornet.org
62	Terpene not specified	–	1,971	MS	–
63	2-Hexenoic acid (E)	Fruity, sweet, warm, herbal	1,981	MS + LRI	flavornet.org
64	*cis*-Lanceol	–	1,995	MS	–
65	*trans*-Nerolidol	Floral, green, citrus, woody	2,035	MS	–
66	Caprylic acid	Fatty, waxy, rancid, oily	2,062	MS + LRI	Ferreira et al., 2001
67	Eugenol	Spicy	2,157	MS + LRI	Cirlini et al., 2016

**Table 2:** Concentrations (μg/mL) of volatile compounds identify in elderberry juice (control) and elderberry juices added with *L. casei* 2057, 2306, 2240, 2246 and the respective cell-free extracts (CFE).

**Compounds**	**VAR**	**Control**	**Zero-growth**		**CFE**				**Growth**	
			**2057**	**2306**	**2057**	**2240**	**2246**	**2306**	**2240**	**2246**
**KETONES**										
Acetone	V-K1	0.047 ± 0.018a, b	0.045 ± 0.034a, b	0.032 ± 0.006a, b	0.152 ± 0.038c	0.072 ± 0.011a, b	0.093 ± 0.000*b, c*	0.053 ± 0.000a, b	0.019 ± 0.002a	0.016 ± 0.001a, b
Diacetyl	V-K2	0.007 ± 0.006a	0.443 ± 0.047*d*	0.37 ± 0.016c	0.011 ± 0.005a	0.004 ± 0.003a	0.003 ± 0.001a	0.003 ± 0.000a	0.279 ± 0.007b	0.255 ± 0.007b
Acetoin	V-K3	0.021 ± 0.001a	0.168 ± 0.103a, b	0.116 ± 0.037a	0.057 ± 0.018a	0.026 ± 0.009a	0.026 ± 0.000a	0.015 ± 0.000a	0.316 ± 0.035b	0.092 ± 0.002a
2,3-Octanedione	V-K4	0.003 ± 0.000a, b, c	0.001 ± 0.001a	0.001 ± 0.001a, b	0.006 ± 0.002c	0.005 ± 0.001b, c	0.004 ± 0.002a, b, c	0.004 ± 0.000a, b, c	0.004 ± 0.000a, b, c	0.001 ± 0.000a, b
Sulcatone	V-K5	0.003 ± 0.003a	0.002 ± 0.003a	0.003 ± 0.001a	n.d.	n.d.	0.001 ± 0.001a	n.d.	0.005 ± 0.001a	0.004 ± 0.001a
Herbal ketone	V-K6	n.d.	0.001 ± 0.001a	0.01 ± 0.011a	n.d.	n.d.	n.d.	n.d.	n.d.	n.d.
2-Undecanone	V-K7	0.001 ± 0.001a	0.003 ± 0.004a	0.002 ± 0.002a	n.d.	n.d.	0.001 ± 0.001a	n.d.	0.002 ± 0.000a	0.002 ± 0.000a
2-Tridecanone	V-K8	n.d.	0.005 ± 0.005a	0.005 ± 0.006a	n.d.	0.001 ± 0.000a	0.001 ± 0.000a	n.d.	0.003 ± 0.001a	0.001 ± 0.000a
2,3,4,5-Tetramethyl-2-cyclopenten-1-one	V-K9	0.002 ± 0.001a	0.002 ± 0.002a	0.003 ± 0.003a	0.001 ± 0.000a	n.d.	0.001 ± 0.001a	n.d.	0.004 ± 0.000a	0.003 ± 0.001a
**ESTERS**										
Ethyl acetate	V-E1	0.054 ± 0.012b, c	0.013 ± 0.01a	0.013 ± 0.011a	0.098 ± 0.001d	0.077 ± 0.019c, d	0.073 ± 0.002c, d	0.053 ± 0.007a, b, c	0.02 ± 0.000a, b	0.028 ± 0.003a, b
Methyl isovalerate	V-E2	0.012 ± 0.002c, d	0.001 ± 0.001a	0.002 ± 0.001a, b	0.014 ± 0.000d	0.01 ± 0.002c, d	0.01 ± 0.003c, d	0.011 ± 0.001c, d	0.008 ± 0.002a, b, c	0.008 ± 0.001c, d
Ethyl isovalerate	V-E3	0.014 ± 0.005a	0.002 ± 0.002a	0.002 ± 0.000a	0.021 ± 0.014a	0.007 ± 0.003a	0.007 ± 0.000a	0.006 ± 0.000a	0.014 ± 0.000a	0.011 ± 0.002a
Isopentyl acetate	V-E4	0.001 ± 0.001a, b	0.002 ± 0.001a, b	0.003 ± 0.001a, b	0.002 ± 0.002a, b	0.001 ± 0.001a	0.001 ± 0.000a, b	0.001 ± 0.000a, b	0.004 ± 0.001b	0.003 ± 0.000a, b
Isoamyl isovalerate	V-E5	0.002 ± 0.001a	0.001 ± 0.000a	0.001 ± 0a	n.d.	n.d.	0.001 ± 0.000a	n.d.	0.003 ± 0.001a	0.003 ± 0.001a
*(E*)-2-Hexenyl-acetate	V-E6	0.001 ± 0.001a	n.d.	0.002 ± 0.001a	n.d.	n.d.	n.d.	0.001 ± 0.000a	0.003 ± 0.001a	0.001 ± 0.000a
Methyl salicylate	V-E7	0.009 ± 0.005a	0.022 ± 0.012a	0.006 ± 0.000a	0.013 ± 0.005a	0.01 ± 0.004a	0.008 ± 0.001a	0.007 ± 0.001a	0.017 ± 0.001a	0.014 ± 0.000a
Phenethyl acetate	V-E8	0.004 ± 0.004a	0.005 ± 0.004a	0.006 ± 0.008a	0.001 ± 0.002a	0.002 ± 0.000a	0.001 ± 0.000a	0.001 ± 0.001a	0.007 ± 0.000a	0.007 ± 0.001a
Alcohols										
Ethanol	V-AL1	0.103 ± 0.011a	0.098 ± 0.047a	0.081 ± 0.03a	0.291 ± 0.11b	0.141 ± 0.031a, b	0.161 ± 0.009a, b	0.093 ± 0.009a	0.077 ± 0.003a	0.075 ± 0.012a
Isoamyl alcohol	V-AL2	0.112 ± 0.013a	0.158 ± 0.135a	0.153 ± 0.1a	0.306 ± 0.114a	0.146 ± 0.048a	0.138 ± 0.000a	0.091 ± 0.003a	0.126 ± 0.004a	0.143 ± 0.027a
2-Penten-1-ol	V-AL3	0.004 ± 0.000a	0.007 ± 0.004a	0.005 ± 0.003a	0.01 ± 0.003a	0.005 ± 0.002a	0.005 ± 0.001a	0.003 ± 0.000a	0.005 ± 0.000a	0.003 ± 0.000a
Hexanol	V-AL4	0.069 ± 0.004a	1.968 ± 1.072b	1.47 ± 0.474a, b	0.176 ± 0.067a	0.094 ± 0.027a	0.092 ± 0.000a	0.061 ± 0.002a	0.027 ± 0.001a	0.062 ± 0.002a
(*E*)-3-Hexen-1-ol	V-AL5	0.008 ± 0.007a, b	0.067 ± 0.033c	0.049 ± 0.005c, d	0.002 ± 0.001a	0.001 ± 0.000a	0.001 ± 0.001a	0.001 ± 0.000a	0.003 ± 0.001a	0.002 ± 0.000a
(*Z*)-3-Hexen-1-ol	V-AL6	0.043 ± 0.006a	0.422 ± 0.237b	0.294 ± 0.044a, b	0.1 ± 0.025a	0.057 ± 0.017a	0.059 ± 0.002a	0.036 ± 0.001a	0.038 ± 0.004a	0.042 ± 0.002a
3-Octanol	V-AL7	n.d.	0.002 ± 0.001c	0.001 ± 0.000a, b	n.d. a	0.001 ± 0.001a	n.d.	n.d.	0.002 ± 0.000c, d	0.001 ± 0.000*a, b, c*
(*E*)-2-Hexen-1-ol	V-AL8	0.012 ± 0.008a	1.763 ± 0.965b	1.361 ± 0.399a, b	0.01 ± 0.003a	0.006 ± 0.002a	0.006 ± 0.000a	0.004 ± 0.000a	0.05 ± 0.001a	0.017 ± 0.001a
1-Octen-3-ol	V-AL9	0.011 ± 0.003a	0.02 ± 0.01a	0.015 ± 0.001a	0.021 ± 0.007a	0.012 ± 0.004a	0.011 ± 0.000a	0.007 ± 0.000a	0.018 ± 0.001a	0.011 ± 0a
Heptanol	V-AL10	0.002 ± 0.000a	0.015 ± 0.005c	0.009 ± 0.000c, d	0.005 ± 0.001a, b	0.002 ± 0.000a	0.003 ± 0.000a	0.002 ± 0.001a	0.001 ± 0.000a	0.001 ± 0.000a
Octanol	V-AL11	0.005 ± 0.002a	0.013 ± 0.003b	0.007 ± 0.002a, b	0.006 ± 0.002a, b	0.004 ± 0.001a	0.004 ± 0.001a	0.003 ± 0.000a	0.001 ± 0.000a	0.003 ± 0.000a
6-Methyl-heptanol	V-AL12	0.005 ± 0.000a	0.025 ± 0.007c	0.016 ± 0.001c, d	0.011 ± 0.003a, b	n.d.	0.005 ± 0.000a, b	0.004 ± 0.000a	0.003 ± 0.001a	0.005 ± 0.000a
Nonanol	V-AL13	0.004 ± 0.003a	0.003 ± 0.001a	0.02 ± 0.027a	0.002 ± 0.001a	0.002 ± 0.001a	0.002 ± 0.000a	0.001 ± 0.000a	0.007 ± 0.004a	0.013 ± 0.003a
2-Furanmethanol	V-AL14	0.019 ± 0.001a	0.079 ± 0.031b	0.05 ± 0.008a, b	0.049 ± 0.016a, b	0.025 ± 0.007a	0.023 ± 0.000a	0.018 ± 0.002a	0.019 ± 0.001a	0.019 ± 0.002a
2- Phenylmethanol	V-AL15	0.037 ± 0.021a	0.067 ± 0.027a	0.047 ± 0.024a	0.048 ± 0.014a	0.029 ± 0.008a	0.028 ± 0.001a	0.022 ± 0.004a	0.054 ± 0.001a	0.052 ± 0.002a
2-Phenylethanol	V-AL16	0.124 ± 0.085a	0.196 ± 0.062a	0.194 ± 0.031a	0.114 ± 0.031a	0.066 ± 0.022a	0.064 ± 0.003a	0.045 ± 0.005a	0.202 ± 0.008a	0.227 ± 0.014a
Dodecanol	V-AL17	n.d.	0.003 ± 0.004a	0.001 ± 0.001a	n.d.	0.001 ± 0.001a	n.d.	n.d.	0.001 ± 0.000a	0.001 ± 0.000a
6-Methyl-octanol	V-AL18	0.004 ± 0.001a	0.025 ± 0.01b	0.024 ± 0.003b	0.017 ± 0.006a, b	0.009 ± 0.003a, b	0.008 ± 0a, b	0.007 ± 0.001a	0.003 ± 0.001a	0.004 ± 0.002a
**TERPENES AND NORISOPRENOIDS**										
Terpene not specified	V-T1	0.002 ± 0.001a	0.001 ± 0.001a	0.001 ± 0.001a	0.002 ± 0.001a	n.d.	0.001 ± 0.000a	n.d.	0.002 ± 0.000a	0.002 ± 0.000a
3-Carene	V-T2	0.004 ± 0.002a	0.002 ± 0.001a	0.002 ± 0.002a	0.005 ± 0.002a	0.002 ± 0.000a	0.002 ± 0.000a	0.002 ± 0.001a	0.004 ± 0.001a	0.004 ± 0.000a
Myrcene	V-T3	0.001 ± 0.001a, b	0.001 ± 0.000a, b	0.001 ± 0.000a, b	0.001 ± 0.000a, b	n.d.	0.001 ± 0.000a	n.d.	0.003 ± 0.001c, d	0.004 ± 0.000c
Limonene	V-T4	0.215 ± 0.061a	0.28 ± 0.242a	0.501 ± 0.062a	0.323 ± 0.164a	0.145 ± 0.034a	0.138 ± 0.001a	0.123 ± 0.009a	0.216 ± 0.103a	0.235 ± 0.097a
Eucalyptol	V-T5	0.005 ± 0.005a	0.005 ± 0.003a	0.003 ± 0.002a	0.002 ± 0.001a	0.001 ± 0.000a	0.001 ± 0.000a	0.001 ± 0.000a	0.007 ± 0.002a	0.003 ± 0.000a
γ-terpinene	V-T6	0.006 ± 0.001a	0.004 ± 0.005a	0.004 ± 0.004a	0.01 ± 0.007a	0.006 ± 0.002a	0.005 ± 0.001a	0.005 ± 0.001a	0.014 ± 0.001a	0.018 ± 0.015a
*m*-cymene	V-T7	0.011 ± 0.001a	0.013 ± 0.003a	0.012 ± 0.003a	0.02 ± 0.016a	0.006 ± 0.001a	0.006 ± 0.000a	0.005 ± 0.001a	0.014 ± 0.001a	0.016 ± 0.001a
Terpinolene	V-T8	0.005 ± 0.003a	0.007 ± 0.001a	0.006 ± 0.003a	0.003 ± 0.000a	0.002 ± 0.001a	0.002 ± 0.000a	0.002 ± 0.000a	0.012 ± 0.007a, b	0.021 ± 0.003b
α-Ionene	V-T9	0.002 ± 0.001a	0.007 ± 0.001a	0.004 ± 0.005a	0.002 ± 0.001a	0.001 ± 0.000a	0.002 ± 0.000a	0.001 ± 0.000a	0.004 ± 0.001a	0.004 ± 0.001a
*cis*-Linalool oxide	V-T10	0.003 ± 0.001a	0.007 ± 0.003a	0.004 ± 0.002a	0.004 ± 0.001a	0.003 ± 0.001a	0.006 ± 0.002a	0.003 ± 0.002a	0.004 ± 0.002a	0.002 ± 0.000a
β-Linalool	V-T11	0.198 ± 0.009a, b	0.941 ± 0.347c	0.713 ± 0.049c, d	0.561 ± 0.216a, b, c	0.301 ± 0.075a, b	0.291 ± 0.002a, b	0.202 ± 0.013a, b	0.156 ± 0.007a	0.151 ± 0.011a
β-Caryophyllene	V-T12	0.003 ± 0.002a	0.002 ± 0.002a	0.001 ± 0.000a	0.001 ± 0.000a	n.d. a	0.001 ± 0.000a	0.001 ± 0.000a	0.005 ± 0.001a	0.006 ± 0.001a
α-Terpineol	V-T13	0.039 ± 0.007a, b	0.149 ± 0.003c	0.138 ± 0.002c	0.069 ± 0.02b	0.041 ± 0.004a, b	0.037 ± 0.001a, b	0.029 ± 0.011a	0.046 ± 0.001a, b	0.044 ± 0.004a, b
Cadina-1(10)-4-diene	V-T14	0.003 ± 0.002a	0.004 ± 0.003a	0.002 ± 0.002a	0.001 ± 0.000a	0.001 ± 0.000a	0.001 ± 0.000a	0.001 ± 0.000a	0.005 ± 0.000a	0.004 ± 0.000a
β-damascenone “like”	V-T15	0.003 ± 0.000a	0.015 ± 0.002b	0.013 ± 0.002b	0.007 ± 0.002a	0.005 ± 0.000a	0.004 ± 0.001a	0.003 ± 0.001a	0.004 ± 0.000a	0.003 ± 0.000a
β-citronellol *(R*)	V-T16	0.003 ± 0.003a	0.015 ± 0.016a	0.001 ± 0.001a	0.001 ± 0.001a	0.001 ± 0.000a	0.001 ± 0.000a	0.001 ± 0.001a	0.004 ± 0.001a	0.003 ± 0.000a
Terpene not specified	V-T17	0.002 ± 0.002a, b	0.002 ± 0.001a, b	0.001 ± 0.000a	n.d. a	0.001 ± 0.000a	0.001 ± 0.000a	n.d. a	0.008 ± 0.001c, d	0.009 ± 0.004c
*cis*-Geraniol	V-T18	0.002 ± 0.001a	0.015 ± 0.004c	0.012 ± 0.004c, d	0.005 ± 0.001a, b	0.004 ± 0.000a, b	0.003 ± 0.001a	0.002 ± 0.000a	0.006 ± 0.002a	0.003 ± 0.000a
β-damascenone	V-T19	0.066 ± 0.024a	0.351 ± 0.036b	0.268 ± 0.004b	0.11 ± 0.034a	0.067 ± 0.016a	0.062 ± 0.002a	0.047 ± 0.006a	0.095 ± 0.007a	0.097 ± 0.003a
*trans*-Geraniol	V-T20	0.005 ± 0.001a	0.035 ± 0.001d	0.026 ± 0.003c	0.01 ± 0.004a, b	0.006 ± 0.001a, b	0.006 ± 0.001a, b	0.005 ± 0.002a	0.014 ± 0.002b	0.007 ± 0.000a, b
Terpene not specified	V-T21	n.d.	0.002 ± 0.001a	0.001 ± 0.001	n.d.	n.d.	n.d.	n.d.	0.001 ± 0.000a	0.001 ± 0.000a
*cis*-Lanceol	V-T22	0.001 ± 0.001a	0.003 ± 0.004a	0.002 ± 0.000a	n.d.	0.001 ± 0.000a	0.001 ± 0.000a	n.d.	0.002 ± 0.001a	0.002 ± 0.000a
*trans*-Nerolidol	V-T23	n.d.	0.002 ± 0.002a	0.001 ± 0.001a	n.d.	0.001 ± 0.000a	0.001 ± 0.001a	0.001 ± 0.000a	0.001 ± 0.000a	n.d.
Eugenol	V-T24	0.008 ± 0.008a	0.014 ± 0.006a	0.008 ± 0.003a	0.002 ± 0.001a	0.001 ± 0.000a	0.001 ± 0.001a	0.001 ± 0.000a	0.094 ± 0.004c	0.042 ± 0.001b
Hotrienol	V-T25	0.001 ± 0.000a	0.004 ± 0.001a	0.002 ± 0.002a	0.001 ± 0.002a	n.d.	0.001 ± 0.000a	n.d.	0.001 ± 0.000a	0.002 ± 0.002a
Dihydro-citronellol	V-T26	0.008 ± 0.001a	0.042 ± 0.018b	0.025 ± 0.002a, b	0.022 ± 0.009a, b	0.011 ± 0.004a	0.009 ± 0.001a	0.007 ± 0.001a	0.007 ± 0.000a	0.006 ± 0.000a
**ACIDS**										
Acetic acid	V-AC1	0.007 ± 0.005a	0.001 ± 0.000a	0.002 ± 0.002a	0.02 ± 0.001b	n.d. a	n.d.a	n.d. a	0.123 ± 0.001d	0.096 ± 0.002c
Isovaleric acid	V-AC2	0.307 ± 0.095a	0.007 ± 0.004a	0.067 ± 0.023a	0.676 ± 0.153b	0.275 ± 0.124a	0.267 ± 0.017a	0.159 ± 0.013a	0.289 ± 0.018a	0.308 ± 0.028a
Caproic acid	V-AC3	0.009 ± 0.009a	0.001 ± 0.000a	0.001 ± 0.000a	n.d.	n.d.	n.d.	n.d.	0.043 ± 0.003b	0.032 ± 0.004b
Caproic acid like	V-AC4	n.d.	0.001 ± 0.000a	n.d.	n.d.	0.001 ± 0.001a	n.d.	n.d.	0.001 ± 0.000a	0.001 ± 0.000a
2-Hexenoic acid *(E*)	V-AC5	n.d.	0.002 ± 0.001b	n.d.	n.d.	0.001 ± 0.000a, b	n.d.	n.d.	0.012 ± 0.001c	0.012 ± 0.000c
Caprylic acid	V-AC6	0.001 ± 0.001a	0.001 ± 0.001a	n.d.	n.d.	n.d.	0.001 ± 0.001a	n.d.	0.006 ± 0.001b	0.008 ± 0.001b

*For each compound a specific variable (VAR), used in statistical and graphical analyses, was assigned*.

*Values are reported as mean , standard deviations, values followed by different letters within a row are significantly different (Tukey's HSD, p < 0.05)*.

*n.d., not detected in the adopted conditions*.

**Table 3:** Mass spectral characteristics of (poly)phenolic compounds detected in elderberry juices added with viable cells and the respective cell-free extracts.

**Compound**	**Rt (min)**	**[M^−^H]^−^(*m/z*)**	**MS^2^ (*m/z*)**	**MS^3^ (*m/z*)**
3-*O*-caffeoylquinic acid	3.80	353	**191**, 179, 135, 173	85, 127, 93, 111, 173
5-*O*-caffeoylquinic acid	4.45	353	**191**, 179	173, 127, 85, 93, 111
4-*O*-caffeoylquinic acid	4.57	353	**173**, 179, 191	–
Coumaroylquinic acid (1)	4.40	337	**163**, 191, 173	–
Coumaroylquinic acid (2)	5.25	337	**191**, 173, 163	–
Caffeic acid	4.90	179	**135**	–
Protocatechuic acid	3.45	153	**109**	–
Phenyllactic acid	6.03	165	**147**,119	–
*p*-hydroxyphenyllactic acid	3.99	181	**163**, 135	–
Luteolin	7.94	285	241, 243, 199, 175, 217, 151	–
Quercetin	7.92	301	179, 151, 273, 239,193	–
Kaempferol	8.99	285	151, 257, 229, 213, 243, 241, 239, 185, 169	–
Isorhamnetin	9.20	315	300, 247	–
Quercetin-3-*O*-glucoside	5.92	463	**301**	179, 151, 273, 239, 193
Kaempferol-*O*-rutinoside	6.17	593	**285**	257, 267, 241, 239, 229, 213, 199, 197, 195, 223, 163, 151
Quercetin-*O*-rutinoside-*O*-hexoside	5.59	771	**301**	179, 151
Isorhamnetin-*O*-hexoside	6.55	477	**314**, 315, 357, 151, 179	300, 271, 285, 286, 299, 275, 243
Isorhamnetin-*O*-rutinoside	6.29	623	**315**, 300, 271, 255	300, 287, 271
Quercetin-3-*O*-rutinoside	5.94	609	**301**, 343	179, 151, 193, 257
Cyanidin-3-*O*-sambubioside	3.43	581	**287**, 449	287, 231, 241, 259, 213
Cyanidin-3-*O*-glucoside	4.38	449	**287**	–
Cyanidin-3-*O*-rutinoside	4.47	595	**287**, 449	–
Cyanidin-3-*O*-sambubioside- hexoside	3.74	743	**287**, 449, 581	287, 259, 231, 213, 241

*Fragment ions are listed in order of their relative abundances. MS^2^ fragment ions reported in bold are those subjected to MS^3^ experiment and used as quantifier ions*.

**Table 4:** Concentration (μg/mL) of (poly)phenolic compounds identified in elderberry juices added with *L. casei* 2057, 2306, 2240, 2246 and the respective cell-free extracts (CFE).

**Compounds**	**VAR**	**Control**	**Zero-growth**		**CFE**				**Growth**	
			**2057**	**2306**	**2057**	**2240**	**2246**	**2306**	**2240**	**2246**
**HYDROXYCINNAMIC ACIDS**										
3-*O*-caffeoylquinic acid	PF-HA1	6.472 ± 0.460a	8.614 ± 0.648c, d	6.449 ± 0.326a	8.994 ± 0.257c, d	7.987 ± 0.315c, d	7.649 ± 0.619a, b, c	9.931 ± 0.957d	6.96 ± 0.109a, b	6.64 ± 0.402a, b
5-*O*-caffeoylquinic acid	PF-HA2	5.605 ± 0.105a	16.116 ± 0.429*f*	6.439 ± 0.411a	10.23 ± 0.129d	9.659 ± 0.454c, d	9.263 ± 0.467*c*	11.569 ± 0.242*e*	7.514 ± 0.137*b*	6.227 ± 0.121a
4-*O*-caffeoylquinic acid	PF-HA3	2.808 ± 0.153a	3.92 ± 0.277c, d	3.108 ± 0.087a, b	4.913 ± 0.262d	4.62 ± 0.152c, d	4.559 ± 0.351c, d	5.326 ± 0.645d	3.239 ± 0.198a, b	3.048 ± 0.311a, b
Coumaroylquinic acid (1)	PF-HA4	0.008 ± 0*b*	0.004 ± 0.001a	0.01 ± 0.001c, d	0.013 ± 0.001*d, e*	0.012 ± 0.001c, d	0.01 ± 0.002c, d	0.015 ± 0.002*e*	0.009 ± 0c, d	0.01 ± 0c, d
Coumaroylquinic acid (2)	PF-HA5	0.032 ± 0.005a	0.037 ± 0.005a, b	0.032 ± 0.002a	0.047 ± 0.005c, d	0.046 ± 0.001c, d	0.046 ± 0.001c, d	0.053 ± 0.005*c*	0.031 ± 0.002a	0.034 ± 0.003a
Caffeic acid	PF-HA6	1.692 ± 0.056*b*	2.21 ± 0.045*c*	1.656 ± 0.086*b*	1.41 ± 0.028a	1.326 ± 0.064a	1.316 ± 0.081a	1.475 ± 0.037a	1.745 ± 0.049*b*	1.688 ± 0.01*b*
Total	–	16.617 ± 0.613a	30.889 ± 1.202*e*	17.684 ± 0.734a, b	25.599 ± 0.389c, d	23.643 ± 0.829*c*	22.838 ± 1.426*c*	28.36 ± 1.873*d, e*	19.488 ± 0.181*b*	17.637 ± 0.573a, b
**HYDROXYBENZOIC ACID**										
Protocatechuic acid	PF-HA7	9.077 ± 0.367c, d	7.847 ± 0.183a, b	9.212 ± 0.157*c*	8.234 ± 0.084a, b, c	7.764 ± 0.424a	7.608 ± 0.178a	9.146 ± 1.031*c*	9.463 ± 0.09c, d	10.509 ± 0.007*d*
**PHENYLLACTIC ACIDS**										
Phenyllactic acid	PF-PLA1	1.452 ± 0.126*b*	0.881 ± 0.033a	2.453 ± 0.105*c*	0.643 ± 0.017a	0.62 ± 0.007a	0.886 ± 0.077a	0.73 ± 0.063a	6.082 ± 0.353d	2.67 ± 0.05*c*
*p*-hydroxyphenyllactic acid	PF-PLA2	1.093 ± 0.034c, d	1.361 ± 0.079*c*	3.735 ± 0.177d	0.433 ± 0.039a	0.44 ± 0.116a	0.768 ± 0.069a, b	0.496 ± 0.112a	9.101 ± 0.392*e*	3.809 ± 0.223*d*
Total	–	2.544 ± 0.144*c*	2.236 ± 0.111*c*	6.173 ± 0.256d	1.072 ± 0.041a, b	1.056 ± 0.12a	1.648 ± 0.146a, b	1.222 ± 0.168a, b	15.146 ± 0.378*e*	6.463 ± 0.263*d*
**FLAVONE**										
Luteolin	PF-FF	1.359 ± 0.058d, e	0.919 ± 0.055a	1.374 ± 0.066d, e	1.135 ± 0.036c, d	1.016 ± 0.053a, b	1.029 ± 0.02a, b	1.2250.085 c,d	1.4480.014 e	1.2940.085 c,d,e
**FLAVONOLS**										
Quercetin	PF-F1	7.034 ± 2.428a	14.424 ± 2.127a	9.148 ± 1.039a	13.184 ± 7.115a	9.292 ± 8.143a	11.375 ± 4.786a	18.473 ± 2.276a	9.221 ± 1.621a	6.506 ± 2.742a
Kaempferol	PF-F2	0.466 ± 0.101a	0.516 ± 0.053a	0.458 ± 0.044a	1.441 ± 0.06*c*	1.201 ± 0.06b	1.138 ± 0.092b	1.638 ± 0.115*c*	0.624 ± 0.029a	0.48 ± 0.059a
Isorhamnetin	PF-F3	0.078 ± 0.003a	0.116 ± 0.01*c*	0.082 ± 0.003a, b	0.163 ± 0.003*d, e*	0.15 ± 0.008d	0.144 ± 0.008d	0.177 ± 0.015*e*	0.1 ± 0.002c, d	0.078 ± 0.002a
Total	–	7.578 ± 2.325a	15.057+2.135 a,b	9.688 ± 0.996a, b	14.787 ± 7.132a, b	10.644 ± 8.147a, b	12.656 ± 4.885a, b	20.288 ± 2.405b	9.945 ± 1.599a, b	7.064 ± 2.747a
**FLAVONOL GLYCOSIDES**										
Quercetin-3-*O*-glucoside	PF-GF1	13.276 ± 1.223a	23.811 ± 1.819d	13.395 ± 1.496a	21.477 ± 0.804c, d	20.884 ± 0.394c	20.845 ± 1.66c	24.762 ± 1.302b	15.595 ± 0.567b	14.58 ± 0.455a, b
Kaempferol-*O*-rutinoside	PF-GF2	0.434 ± 0.049a	0.447 ± 0.031a, b	0.423 ± 0.024a	0.614 ± 0.02c, d	0.546 ± 0.044c, d	0.544 ± 0.028c, d	0.68 ± 0.049d	0.472 ± 0.022a, b	0.457 ± 0.031a, b
Quercetin-*O*-rutinoside-*O*-hexoside	PF-GF3	0.004 ± 0.001a	0.016 ± 0.001b	0.005 ± 0a	0.006 ± 0.001a	0.006 ± 0.001a	0.006 ± 0.001a	0.006 ± 0.002a	0.008 ± 0.001a	0.005 ± 0.001a
Isorhamnetin-*O*-hexoside	PF-GF4	0.023 ± 0.003a, b	0.017 ± 0.005a	0.024 ± 0.001a, b	0.031 ± 0b	0.033 ± 0.005b	0.03 ± 0.001b	0.045 ± 0.006c	0.024 ± 0.002a, b	0.025 ± 0.003a, b
Isorhamnetin-*O*-rutinoside	PF-GF5	0.067 ± 0.005a	0.142 ± 0.02c, d	0.069 ± 0.003a	0.147 ± 0.005c, d	0.141 ± 0.008c, d	0.129 ± 0.017b	0.166 ± 0.019c	0.079 ± 0.002a	0.071 ± 0.006a
Quercetin-3-*O*-rutinoside	PF-GF6	29.98 ± 0.654a	63.319 ± 3.345d	31.389 ± 2.938a, b	48.582 ± 1.955c	43.937 ± 2.662c, d	45.226 ± 4.339c	52.34 ± 3.513a, b	35.664 ± 3.999a, b	32.237 ± 3.297a, b
Total	–	35.737 ± 8.274a	87.752 ± 5.18d	45.305 ± 4.008a, b	70.858 ± 1.439c	65.547 ± 2.245c, d	66.779 ± 3.786c, d	77.998 ± 2.175c, d	51.842 ± 4.143b	47.375 ± 3.709a, b
**ANTHOCYANINS**										
Cyanidin-3-*O*-sambubioside	PF-A1	6.318 ± 3.097a	69.114 ± 7.223b	6.537 ± 0.666a	7.656 ± 0.652a	6.042 ± 0.218a	5.762 ± 0.726a	8.019 ± 0.666a	5.17 ± 0.271a	6.263 ± 0.502a
Cyanidin-3-*O*-glucoside	PF-A2	2.772 ± 0.543a	44.524 ± 1.833b	2.257 ± 0.09a	3.319 ± 0.158a	2.842 ± 0.097a	2.809 ± 0.31a	3.46 ± 0.132a	2.927 ± 0.121a	2.122 ± 0.065a
Cyanidin-3-*O*-rutinoside	PF-A3	0.096 ± 0.007a	1.146 ± 0.075b	0.042 ± 0.004a	0.08 ± 0.006a	0.072 ± 0.005a	0.074 ± 0.006a	0.087 ± 0.004a	0.071 ± 0.004a	0.036 ± 0.002a
Cyanidin-3-*O*-sambubioside- hexoside	PF-A4	0.436 ± 0.308a	0.785 ± 0.324c	0.675 ± 0.072c, d	0.483 ± 0.037a, b, c	0.377 ± 0.03a, b	0.339 ± 0.053a, b	0.501 ± 0.034a, b, c	0.447 ± 0.014a, b, c	0.744 ± 0.04*c*
Total	–	9.516 ± 4.316a	115.57 ± 8.584b	9.51 ± 0.827a	11.538 ± 0.816a	9.333 ± 0.221a	8.983 ± 1.072a	12.068 ± 0.791a	8.615 ± 0.398a	9.166 ± 0.589a

*For each compound a specific variable (VAR), used in statistical and graphical analyses, was assigned. Values are reported as mean ± standard deviations, values followed by different letters within a row are significantly different (Tukey's HSD, p < 0.05)*.

Elderberry juices added with viable cells and CFE were grouped in three distinct cluster, separated from the control (Figure 1A). Samples where bacterial growth occurred were separated from the control in the upper right quadrant of PCA, strongly positively associated with all the acids (acetic acid, caproic acid, 2-hexenoic acid (E) acid, caprilic acid), isoamyl isovalerate and two terpenes.

On the other hand, the variables with strongly negative values on the first component (quercetin, ethyl acetate, methyl isovalerate) were determinant to group the elderberry juices added with CFE.

Interestingly, elderberry juice added with *L. casei* showing zero-growth appeared well separated from the control in lower right quadrant of PCA, associated with variables with positive values on component 1 and negative value on component 2, as many terpenes and alcohols.

**Figure 2:**
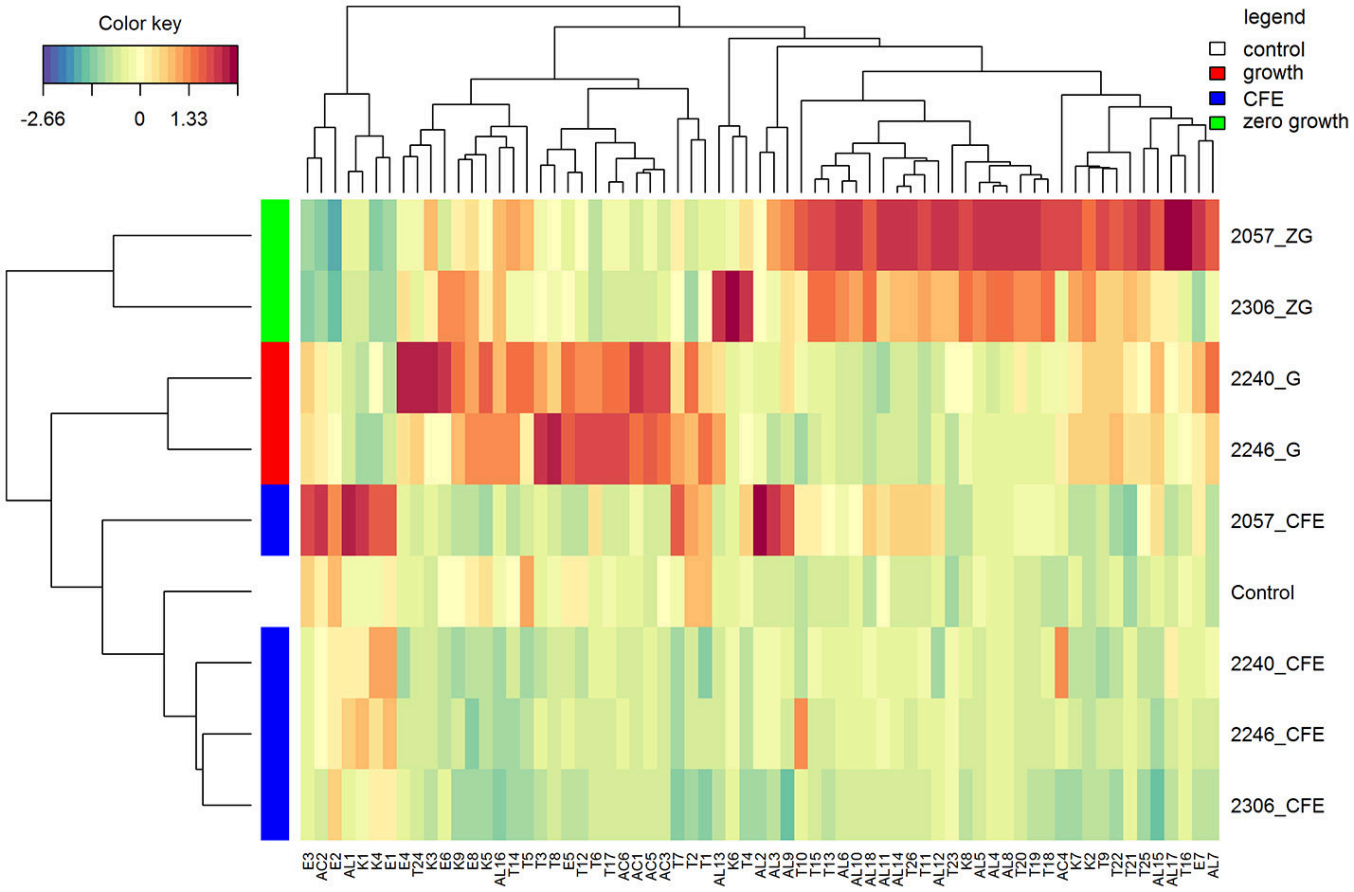
Hierarchical clustering analysis and heatmap visualization of the volatile profiles of the elderberry added with zero growth (ZG) strains, growing strains (G), or cell-free extracts (CFE), and the unstarted juice (control), based on the Euclidean distance calculated on the amount of each volatile compound. The leftmost column bar is colored according to the growth condition used for the fermentation, as reported in the legend. The color scale represents the scaled abundance of each variable, denoted as d^2^ (squared Euclidean distance), with red indicating high abundance and blue indicating low abundance. The compounds represented in the heat-map are named as in Table 2.

In accordance with the PCA analysis, the heatmap describing the volatile compounds showed a separation among the three type of samples highlighting the peculiarities of specific strains (Figure 2). Indeed, elderberry juice added with CFE of *L. casei* 2057, differently from the other CFE samples and the control, has a profile characterized by a higher production of volatile compounds, especially esters and alcohols. Despite esters were overall less abundant among the samples (Table 2) they were associated to fruit notes contributing positively to the aromatic profile. The heatmap also clearly displays that samples showing either a growth or a zero-growth phenotype were marked by different volatiles, that were mainly acid, esters and ketones for the former, and alcohols and terpenes for the latter. Most of the acids, acetic in particular, increased in juice fermented with *L. casei* growing strains 2240 and 2246 (Table 2), whereas isovaleric acid resulted higher in the juice added with CFE of *L. casei* 2057 compared to the control (*p* < 0.05), where it reached 0.68 ± 0.15 μg/mL.

Moreover, after 48 h of incubation the alcohol content increased, especially in samples with zero-growth, with the highest production found in elderberry juice with *L. casei* 2057 (4.93 ± 0.73 μg/mL) (Table 3). Notably, hexanol (V-AL4), and (*E*) 2- hexen-1-ol (V-AL8), associated with green aromatic notes, were the most abundant and their concentration significantly differs from control (*p* < 0.05). Similarly, terpenes and norisoprenoids, such as β-damascenone, β-linalool and α-terpineol, increased mainly in juice added with zero-growth strains *L. casei* 2057 and *L. casei* 2306 (Table 2).

**Figure 3:**
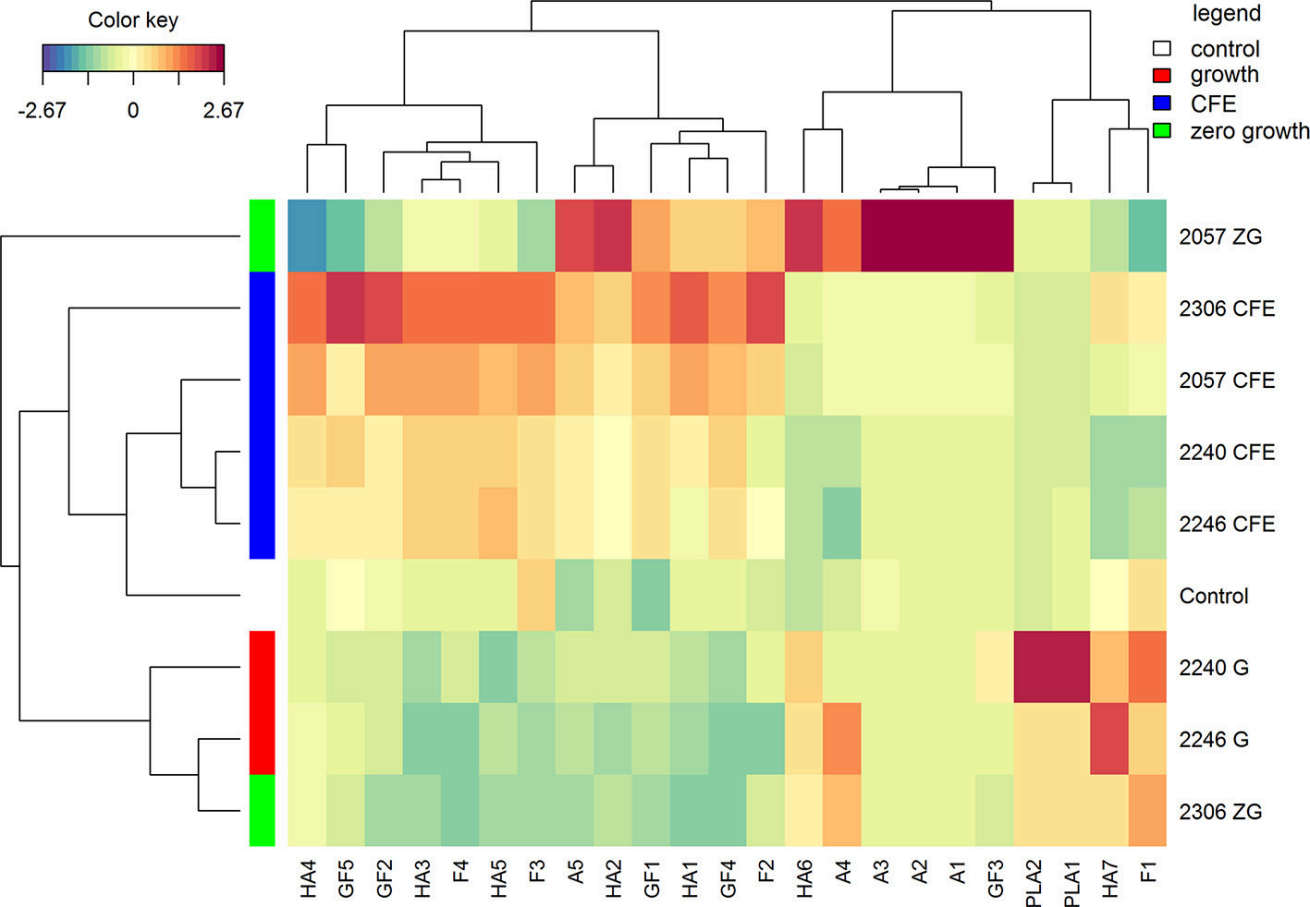
Hierarchical clustering analysis and heatmap visualization of the polyphenolic compounds identified in the elderberry juices added with zero growth (ZG) strains, growing strains (G), or cell-free extracts (CFE), and the unstarted juice (control), based on the Euclidean distance calculated on the amount of each identified polyphenol. The leftmost column bar is colored according to the growth condition used for the fermentation, as reported in the legend. The color scale represents the scaled abundance of each variable, denoted as d^2^ (squared Euclidean distance), with red indicating high abundance and blue indicating low abundance. The compounds represented in the heat-map are named as in Table 4.

The polyphenolic profiles drive a different clusterization of samples in comparison with volatile compounds (Figure 3). CFE and growth strains grouped separately, instead samples with zero-growth showed a different potential. To note, despite the absence of growth, *L. casei* 2057 was able to significantly increase many polyphenolic compounds, especially anthocyanins (Table 4).

### Effect of viable cells and cell-free extracts of *Lactobacillus casei* on polyphenolic profile

The (poly)phenolic profile of samples analyzed was mainly characterized by hydroxycinnamic acids, phenyllactic acids, flavone, flavonols, flavonol glycosides, anthocyanins and hydroxybenzoic acid. Samples shown a different trend of hydroxycinnamic acids. An increase, in comparison with the control, was observed for all the juice added with CFE, but especially in juice added with *L. casei* 2057 zero-growth (Table 4). Conversely, juice fermented with growing *L. casei* showed a reduction in these compounds after 48 h or no differences with unfermented juice (Table 4). About phenyllactic acids only 3 samples have shown differences in comparison to the control and among these, fermentation with 2240 growing strain, lead to the highest concentration of phenyllatic acid (6.08 ± 0.35 μg/mL) and *p*-hydroxyphenyllactic acid (9.10 ± 0.39 μg/mL) (Table 4). Flavonoids identified comprise flavone, flavonols (mainly found as glycosides) and anthocyanins. Total flavonols (not glycosylated), as quercetin, kaempferol and isorhamnetin, resulted highest in the juice added with CFE of *L. casei* 2306 (20.29 ± 2.40 μg/mL) (Table 4). However, a higher concentration of these compounds was observed in their glycosylated forms: quercetin as quercetin-3-*O*-rutinoside, quercetin-3-*O*-glucoside, kaempferol as kaempferol-*O*-rutinoside and isorhamnetin as isorhamnetin-*O*-hexoside or isorhamnetin-*O*-rutinoside. Quercetin-3-*O*-rutinoside resulted the highest especially in juice added with *L. casei* 2057 zero-growth and quercetin-3-*O*-glucoside in CFE of 2306 (both significantly different when compared to the control, *p* < 0.01). The last and the most interesting results were about the anthocyanins (Table 4). Surprisingly, we observed a huge increase of these compounds in juice added with *L. casei* 2057 zero-growth, where their concentration reached 115.57 ± 8.58 μg/mL, in comparison of 9.62 ± 0.54 μg/mL of the control. In particular, the most abundant anthocyanins were cyanidin-3-*O*-sambubioside and cyanidin-3-*O*-glucoside, while cyanidin-3-*O*-rutinoside and cyanidin-*O*-sambubioside-*O*-hexoside were almost negligible.

### Comparison of juices added with viable cells and CFE of *L. casei*

In order to better highlight the differences among juices added with viable cells and CFE we focused on variables that significantly differ among the samples, belonging to the most abundant classes of detected compounds which are alcohols and terpenes for the volatiles and anthocyanins and flavonol glycosides for polyphenols. Moreover, we have considered phenyllactic acids because they are often found in fermented food as a consequence of microbial metabolism and exert an interesting role as antimicrobials.

**Figure 4:**
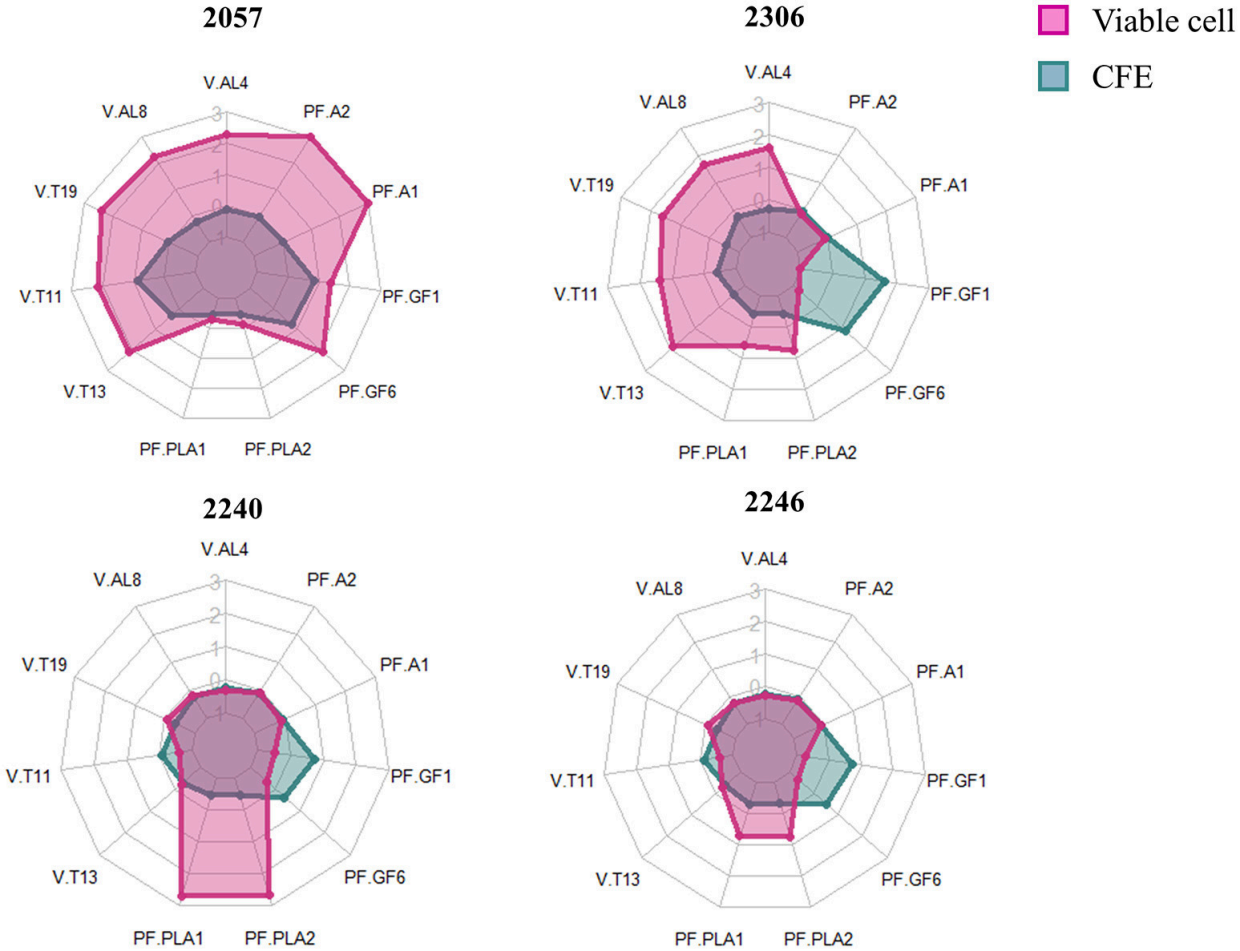
Radarplot of the scaled quantity of the compounds that significantly differ among samples. Radars show the scaled amount of compounds produced by the four strains added to elderberry juice as viable cells (pink) or as cell-free extract (CFE, blue). The abbreviations for volatile compounds (V) and polyphenols (PF) are the following: V.AL4, Hexanol; V.AL8, (E)-2-Hexen-1-ol; V.T19, β-damascenone; V.T11, β-Linalool; V-T13, α-Terpineol; PF.PLA1, DL-phenyllactic acid; PF.PLA2, p-Hydroxyphenyllactic acid; PF-GF6, Rutin; PF.GF1, Quercetin-O-hexoside; PF.A1, Cianidina-O-sambubioside; PF.A2, Cianidina-O-esoside.

The radar plot (Figure 4) shows how viable cells and CFE from the same strain differently impact on the variables considered. Firstly, the viable cells lead to increase the relevant polyphenols and volatiles more than CFE, except for strain 2246. It can also be observed that viable cells of growing strains, in particular the strain 2240, were able to especially increase the content of phenyllactic acids while viable cells of non-growing strains strongly impact on more variables, especially alcohol and terpenes. Finally, the strain 2057, even in absence of growth, shows the greatest impact to increase the polyphenolic content (especially anthocyanins) and the volatile compounds associated with typical aroma of elderberry juice (hexanol, β-linalool, α-terpineol, β-damascenone and (*E*)-2-hexen-1-ol).

## Discussion

The aim of this study was to investigate the changes in volatile compounds and polyphenols in elderberry juice added with viable and CFE of 4 *L. casei* strains.

Beyond the active metabolism of growing cells, it was demonstrated that flavor production occurs when LAB reach a non-growing state (Van de Bunt et al., 2014). Moreover, during the ripening of fermented food, the contribution of microbial intracellular components, released after cells lysis, have a fundamental role in the modulation of aromatic profile (Lortal and Chapot-Chartier, 2005; Bolumar et al., 2006; Styger et al., 2011). So far, no previous work had focused on the addition of cell extracts or viable but non-multiplying bacterial cells into fruit juices and, to the best of our knowledge, only our group did considered the use of LAB specifically in elderberry juice (Ricci et al., 2018). To better describe the different contribution of viable cells and CFE, a commercial pasteurized elderberry juice was used because, even if heat treatments could in part change the chemical composition of the raw material, sterilized/pasteurized juices are the best model systems to carry out a steered fermentation (Gardner et al., 2001).

Juices added with CFE, especially *L. casei* 2057, showed the highest amount of esters, which are formed by the esterification of acids and alcohols (McSweeney, 2004; Smid and Kleerebezem, 2014). They exhibit fruity notes with low threshold concentrations, many times lower than their precursors, (Di Cagno et al., 2009), and for this reason are considered influential even if detected at low levels. The main compounds identified in juice added with CFE, namely ethyl acetate and methyl salicylate, probably derived from the esterification of ethanol with acetic acid and shikimic acid, a metabolite present in elderberry, respectively (Kaack et al., 2005).

Viable cells of *L. casei* strains added to elderberry juice showed two distinct effects, described as bacterial growth, associated to fermentation, and absence of growth, or zero-growth.

The strains showing a growth phenotype (2240 and 2246) elicited an increase in acids production, such as acetic acid, as a result of heterofermentative pathways, in line with other works on lactic acid fermentation of plant matrix (Filannino et al., 2013, 2017). Furthermore, these strains significantly increased the amounts of phenyllactic acids (phenyllactic acid and *p*-hydroxyphenyllactic acid). Phenyllactic acids are naturally found in honey and LAB fermented food (Mu et al., 2012) and can be produced by different species of LAB in a strain-dependent way (Valerio et al., 2004), but, to the best of our knowledge, this is the first time that has been reported for *L. casei*. Originating from the metabolism of aromatic amino acids, phenyllactic acid is produced from degradation of phenylalanine, while *p*-hydroxyphenyllactic acid is produced from degradation of tyrosine (Valerio et al., 2004). Thanks to their antimicrobial activity, in particular against fungi, but also against different species of bacteria (Lavermicocca et al., 2000; Valerio et al., 2009; Cortés-Zavaleta et al., 2014), their use as natural preservative in food has been proposed (Lavermicocca et al., 2000).

Regarding volatile compounds, the strains exhibiting zero-growth lead to the highest increase in their total amount, in particular in those alcohols, terpenes and norisoprenoids which exhibit typical elderberry scent (Kaack et al., 2005; Kaack, 2008).

Among alcohols, 2-hexen-1-ol and hexanol, related with the green elderberry note, were produced in the highest amounts. These compounds are degradation products of linoleic and linolenic acid, found in elderberry seeds (Dulf et al., 2013), which might be formed via the action of lipoxygenase (Sabatini et al., 2008). Their increase had also been previously reported in elderberry juice fermented with *L. plantarum* and *L. rhamnosus* strains (Ricci et al., 2018) and in olives after lactic acid fermentation (Sabatini et al., 2008), but no study reported this ability in non-growing bacteria. Compared with our previous results (Ricci et al., 2018), where growing strains were employed in elderberry juice, non-growing strains highly increase the concentration of alcohols mentioned above (about 1.4–1.6 μg/mL).

Zero-growth strains had also the capacity to significantly increase the concentration of β-damascenone, an important norisoprenoid strongly associated with elderberry scent. The terpenes β-linalool and α-terpineol impart a floral note to elderberry (Kaack et al., 2005) and are positively affected by zero growth strains used in this study. Different works highlighted the increase of these compounds after fermentation with LAB and their relevance as odorous molecules (Ugliano and Moio, 2006; Cañas et al., 2008). Terpenes and norisoprenoids are usually found in nature as glycosides, but some studies reported the ability of microorganisms to produce glycosylases resulting in the release of aglyconic forms (Ugliano and Moio, 2006; Cañas et al., 2008; Di Cagno et al., 2013). Moreover, Belviso et al. (2011) suggested the possibility for some lactic acid bacteria to synthetize *ex novo* specific terpenes as secondary metabolites.

Another interesting potentiality of zero-growth strains was the ability to increase anthocyanins, particularly cyanidin-3-*O*-sambubioside and cyanidin-3-*O*-glucoside, and glycosylated flavonols, especially quercetin-3-*O*-glucoside and quercetin-3-*O*-rutinoside. An increase of cyanidin-3-*O*-glucoside and quercetin-3-*O*-rutinoside during mulberry fermentation was also reported by Kwaw et al. (2018) especially when *L. paracasei* and *L. acidophilus* were used.

Overall, the most interesting sample, considering all the variables, was the elderberry juice added with *L. casei* 2057. Despite the absence of growth, an increase of volatile compounds, especially alcohols, terpenes and norisoprenoids, and polyphenols, in particular anthocyanins, was observed. Even if the literature is not completely conclusive on the topic, mounting evidence from prospective studies, also supported by recent intervention trials, suggests that an increased intake of anthocyanins could result in health benefits in humans, particularly in the cardiovascular framework. For this reason, every strategy that results in increased content of anthocyanins in food, including fermentation, could be considered as advantageous.

The different effects observed with regards to the growth phenotype exhibited by the *L. casei* strain suggests that, in the selection of starters for fruit juice fermentation, the growth capability of certain strains in the substrate should not be the sole selection criterion. This observation is particularly relevant for bacteria used in industrial food fermentation, where bacterial cells persisting in a non-replicating state have an active metabolism with a detectable transcriptional activity and maintain the capability to produce volatile compounds (Ercan et al., 2015; Van Mastrigt et al., 2018). Further studies will be directed to assess if mechanisms involved in the entrance into non-growing state such as toxin-antitoxin systems, recently described in *Lactobacillus casei* group (Klimina et al., 2013; Folli et al., 2017), could be related with changes in the metabolism of polyphenols and aromatic compounds.

## Conclusion

The selection of microbial culture used in food relies on the ability of growth, on the production of aromatic compounds, and on the increase of nutritional value. Nevertheless, some authors are actively debating about zero-growth state as a physiological condition useful to enhance the aromatic features of a product. In this study, the first carried out using non-growing LAB in fruit juices, we have demonstrated that the polyphenolic profile (i.e., anthocyanins) and the typical elderberry aroma (alcohols, terpenes and norisoprenoids) is strongly modulated in absence of growth.

These results may have a significant technological application, since the potential of non-growing *Lactobacillus* cells, able to boost the presence of specific molecules, could lead to develop a microbial collection exploitable in the fruit juice industry.

## Author contributions

CL and VB conceived the idea for the project. AR, MC, and LC conducted the microbiological and chemical analyses. AL conducted the statistical analysis. DD, GG, and EN reviewed results and discussion; AR, AL, VB, and CL wrote the paper. All authors reviewed the manuscript and approved the final version of the manuscript.

### Conflict of interest statement

The authors declare that the research was conducted in the absence of any commercial or financial relationships that could be construed as a potential conflict of interest.
