# Supplementary material for: The Influence of Viable Cells and Cell-Free Extracts of Lactobacillus casei on Volatile Compounds and Polyphenolic Profile of Elderberry Juice
Source: Front Microbiol. 2018 Nov 20;9:2784. doi: 10.3389/fmicb.2018.02784 (PMC6256114; doi:10.3389/fmicb.2018.02784)
Supplement: Supplementary file 1 [file Table_1.DOCX]

**Table S1:** Variables loading on dimensions 1 and 2 of the principal components analysis (PCA), bold characters highlight the ones selected for further PCA analysis, variables abbreviation are as reported in Tables 2 and 4**.**

| **Variable** | **Dim.1** | **Dim.2** |
| --- | --- | --- |
| **V.K1** | 0.563447 | -0.48795 |
| **V.K2** | **-0.9548** | -0.04539 |
| **V.K3** | -0.66065 | 0.27568 |
| **V.K4** | **0.761758** | -0.13358 |
| **V.K5** | -0.65425 | 0.702856 |
| **V.K6** | -0.24407 | -0.15978 |
| **V.K7** | **-0.83106** | -0.02587 |
| **V.K8** | **-0.75184** | -0.21257 |
| **V.K9** | -0.73453 | 0.522276 |
| **V.E1** | **0.854216** | -0.20771 |
| **V.E2** | **0.852292** | 0.242426 |
| **V.E3** | 0.290788 | 0.386463 |
| **V.E4** | -0.56283 | 0.566947 |
| **V.E5** | -0.43298 | **0.817155** |
| **V.E6** | -0.47942 | 0.558809 |
| **V.E7** | -0.62416 | -0.10316 |
| **V.E8** | -0.71134 | 0.484272 |
| **V.AL1** | 0.458011 | -0.36259 |
| **V.AL2** | 0.08589 | -0.30835 |
| **V.AL3** | -0.03394 | -0.55056 |
| **V.AL4** | -0.68335 | -0.61529 |
| **V.AL5** | -0.73578 | -0.56472 |
| **V.AL6** | -0.66456 | -0.6509 |
| **V.AL7** | **-0.81457** | 0.117299 |
| **V.AL8** | -0.71506 | -0.57077 |
| **V.AL9** | -0.4269 | -0.35512 |
| **V.AL10** | -0.64823 | -0.72542 |
| **V.AL11** | -0.52808 | **-0.76548** |
| **V.AL12** | -0.67141 | -0.65335 |
| **V.AL13** | -0.48156 | 0.330624 |
| **V.AL14** | -0.55069 | -0.72782 |
| **V.AL15** | **-0.83171** | 0.04752 |
| **V.AL16** | **-0.88148** | 0.362635 |
| **V.AL17** | -0.67078 | -0.30039 |
| **V.AL18** | -0.45071 | **-0.75148** |
| **V.T1** | -0.2744 | 0.534271 |
| **V.T2** | -0.11052 | 0.640279 |
| **V.T3** | -0.50403 | 0.722857 |
| **V.T4** | -0.51599 | -0.10296 |
| **V.T5** | -0.67765 | 0.372237 |
| **V.T6** | -0.12675 | 0.63994 |
| **V.T7** | -0.39467 | 0.163035 |
| **V.T8** | -0.51298 | 0.628075 |
| **V.T9** | **-0.91543** | -0.02757 |
| **V.T10** | -0.30514 | -0.58717 |
| **V.T11** | -0.52139 | **-0.76421** |
| **V.T12** | -0.50539 | **0.771227** |
| **V.T13** | -0.74935 | -0.5617 |
| **V.T14** | **-0.79226** | 0.475603 |
| **V.T15** | -0.67976 | -0.65671 |
| **V.T16** | -0.64934 | -0.25898 |
| **V.T17** | -0.40526 | **0.772014** |
| **V.T18** | **-0.77309** | -0.53307 |
| **V.T19** | **-0.81999** | -0.53332 |
| **V.T20** | **-0.85574** | -0.47514 |
| **V.T21** | **-0.7732** | -0.14372 |
| **V.T22** | **-0.81149** | 0.118434 |
| **V.T23** | -0.56966 | -0.33173 |
| **V.T24** | -0.44662 | 0.703271 |
| **V.T25** | **-0.8161** | -0.29213 |
| **V.T26** | -0.58015 | -0.71811 |
| **V.AC1** | -0.31562 | **0.806214** |
| **V.AC2** | 0.509637 | 0.238763 |
| **V.AC3** | -0.34639 | **0.860916** |
| **V.AC4** | -0.45909 | -0.37886 |
| **V.AC5** | -0.39421 | **0.763151** |
| **V.AC6** | -0.39567 | **0.769655** |
| **PF.A1** | -0.63245 | -0.62528 |
| **PF.A2** | -0.62784 | -0.62404 |
| **PF.A3** | -0.61647 | -0.61961 |
| **PF.A4** | -0.64413 | -0.16958 |
| **PF.FF** | 0.187578 | -0.45213 |
| **PF.F1** | **0.799588** | -0.33818 |
| **PF.F2** | 0.651078 | -0.5119 |
| **PF.F3** | -0.10697 | **0.753484** |
| **PF.GF1** | 0.322107 | -0.69819 |
| **PF.GF2** | 0.727809 | -0.2629 |
| **PF.GF3** | -0.61536 | -0.52017 |
| **PF.GF4** | **0.766333** | -0.05092 |
| **PF.GF5** | 0.432861 | -0.68325 |
| **PF.GF6** | -0.00901 | **-0.7696** |
| **PF.HA1** | 0.377784 | -0.59009 |
| **PF.HA2** | -0.15049 | **-0.79229** |
| **PF.HA3** | 0.610601 | -0.54231 |
| **PF.HA4** | **0.778952** | 0.073227 |
| **PF.HA5** | 0.678949 | -0.46884 |
| **PF.HA6** | **-0.85903** | -0.12632 |
| **PF.HA7** | -0.2181 | 0.715579 |
| **PF.PLA1** | -0.44415 | 0.73053 |
| **PF.PLA2** | -0.51013 | 0.672023 |
